# Beyond fluid responsiveness: the concept of fluid tolerance and its potential implication in hemodynamic management

**DOI:** 10.5935/2965-2774.20230012-en

**Published:** 2023

**Authors:** Rafael Hortêncio Melo, Mauricio Henrique Claro dos Santos, Fernando José da Silva Ramos

**Affiliations:** 1 Adult Intensive Care Unit, Hospital Municipal Vila Santa Catarina - São Paulo (SP), Brazil; 2 Adult Intensive Care Unit, Hospital Sírio-Libanês - São Paulo (SP), Brazil; 3 Department of Anesthesiology, Pain and Intensive Care, Universidade Federal de São Paulo - São Paulo (SP), Brazil

## INTRODUCTION

Despite the different methodologies and definitions in the literature, the most accepted concept of fluid responsiveness (FR) is an increase in cardiac output greater than 10-15% induced by an increased preload. Thus, volume expansion is the initial measure most frequently used to optimize tissue perfusion in a hemodynamically unstable patient. However, the prevalence of FR in the intensive care unit (ICU) is approximately 50%.^(1)^ Therefore, the indiscriminate administration of fluids to all patients with hemodynamic instability, in addition to not obtaining the possible benefits induced by the increase in cardiac output, has the potential to aggravate organ dysfunctions, since the excess of fluids, represented by the accumulated hydric balance, is an independent factor for an increase ICU length of stay, duration of mechanical ventilation, probability of acute kidney injury and mortality.^(2-4)^

Fluid tolerance (FT), in turn, is the ability of the body to receive an infusion of fluids without progressing to organ dysfunction^(5)^ (Table 1). Possible mechanisms related to the genesis of these disorders include tissue damage in the microcirculation due to an increase in the distance of oxygen diffusion to the cells and a decrease in the number of oxygenated red blood cells due to hemodilution; alterations in the endothelial glycocalyx^(6)^ with altered vascular permeability^(7)^ and greater tissue edema; and increased intraparenchymal pressure in encapsulated organs, such as the liver and kidneys,^(8)^ resulting in lower perfusion pressure and lower tissue blood flow.

**Table 1:** Organ dysfunction induced by fluid overload

Body	Dysfunction
Lungs	Alteration in gas exchange
	Reduction in complacency
	Increased work of breathing
Heart	Conduction disorders
	Change in contractility
	Diastolic dysfunction
Brain	Cognitive dysfunction
	*Delirium*
Kidney	Increased interstitial pressure
	Reduction in renal blood flow
	Decreased glomerular filtration rate
	Uremia
	Retention of salt and water
Liver	Cholestasis
	Dysfunction of hepatic synthesis
Intestine	Ileum
	Malabsorption
Skin	Reduction in the healing process
	Pressure ulcer
	Wound infection

Thus, the combined evaluation of FR and FT is of fundamental importance in the management of hemodynamically unstable patients because the absence of FT, even in a fluid responsive patient, can mitigate any benefit induced by volume expansion and even aggravate or cause new organ dysfunctions.

### Hemodynamic profiles

Considering the presence or absence of FR and FT, there are four hemodynamic profiles:

- A: FR present and FT present.
- B: FR absent and FT present.
- C: FR absent and FT absent.
- D: FR present and FT absent.

Depending on the hemodynamic profile in question, it is possible to individualize, the most appropriate approach for hemodynamic resuscitation for a given hemodynamically unstable patient, with the objective of preventing and/or reversing organ dysfunction (Table 2).

**Table 2:** Management based on hemodynamic profiles of fluid tolerance and fluid responsiveness

Fluid responsiveness	Fluid tolerance	Hemodynamic management
Present	Present	Volume expansion
Absent	Present	Conservative fluid management
Absent	Absent	Resuscitation: diuretics and ultrafiltration
Present	Absent	Early use of vasopressors

The following are basic principles:

- Volume administration should not be performed in the absence of FR (profiles B and C). In the absence of FR and FT (C profile), the need for de-resuscitation should be assessed. Fluid administration in a fluid tolerant patient but without FR criteria (profile B) can lead to the loss of FT, with the risk of new hypervolemia-induced organ dysfunction, thus requiring conservative management with regard to the administration of fluids.

- In the presence of FR, volume administration with the greatest potential benefit and lower risk of inducing or worsening organ dysfunction will be in the concomitant presence of FT (profile A).

- In the presence of FR and absence of FT (profile D), volume administration should take into account the potential for inducting and/or aggravating organ dysfunction, and early initiation of vasoactive drugs should be considered.

### Methods for evaluating fluid tolerance

Fluid tolerance should be evaluated in two different compartments; left, considering the left heart chambers filling pressures and the degree of pulmonary congestion; and right, assessing the right heart chambers filling pressures and the degree of fluid overload in the systemic venous compartment.

Static measures of filling pressure in right and left cardiac chambers, such as central venous pressure (CVP) and pulmonary artery occlusion pressure (PAOP), depend on the interaction between venous return function and ventricular function, and are also influenced by increases in intrathoracic pressure in situations such as pneumothorax, cardiac tamponade and the use of positive end-expiratory pressure (PEEP). Therefore, they have a limited role in the identification of FR and volemic status; however, extremely low values (< 6mmHg) increase the probability of FR. On the other hand, high values (CVP > 12mmHg and PAOP > 18mmHg) may indicate a low FT capacity in certain clinical circumstances and are associated with an increased risk of peripheral edema, ascites, pulmonary edema, renal and hepatic dysfunction. In addition, the measurement of these variables requires the use of invasive devices, such as a central venous catheter for CVP and a pulmonary artery catheter for PAOP.

Hemodynamic monitoring by transpulmonary thermodilution may assist in the evaluation of FT, especially on the left compartment, using variables such as global end-diastolic volume (GEDV), extravascular lung water (EVLW) and the pulmonary vascular permeability index (IPVP), and is useful in the differential diagnosis between inflammatory and hydrostatic alveolar-interstitial syndrome. Despite being a less invasive tool than a pulmonary artery catheter, the insertion of central venous and arterial catheters is required and has high cost limiting its availability in most intensive care units.

**Figure 1:**
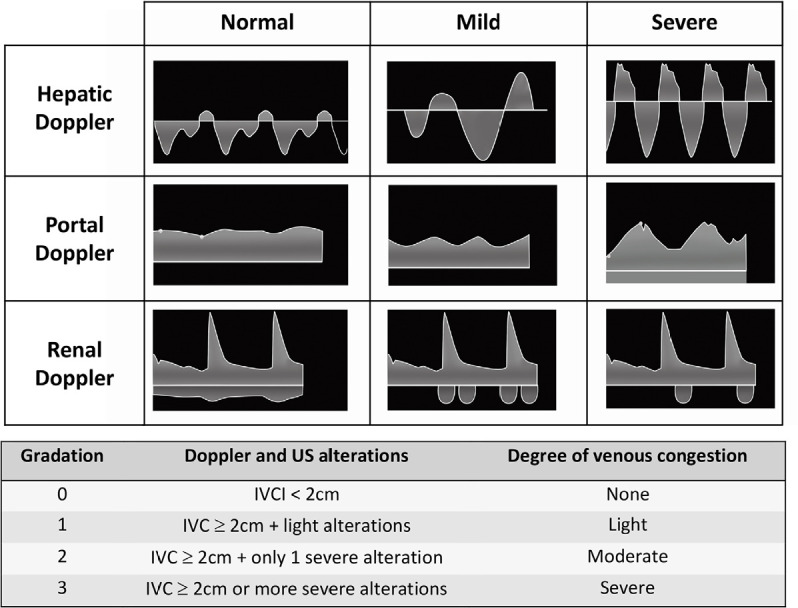
Pulsed Doppler ultrasound pattern of the hepatic, portal and renal interlobar veins for evaluating excess venous congestion.

**Figure 2:**
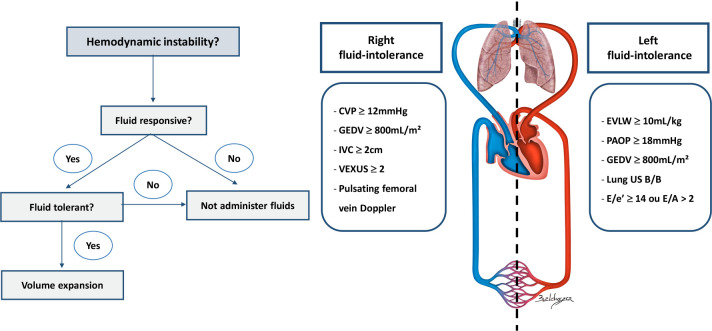
Hemodynamic evaluation of fluid responsiveness and fluid tolerance for decisions on volume expansion.

Point-of-care ultrasonography is becoming increasingly available in ICUs, with the potential for wide application for evaluation of both left and right FT. The estimation of EVLW using lung ultrasound^(9)^ and the evaluation of left ventricular filling pressures, as assessed by the relationship between the E wave and the A wave of the transmitral flow on pulsed Doppler and the E wave over the E’ wave on tissue Doppler, help in the evaluation of left FT. Venous excess ultrasound score (VExUS) takes into account the diameter of the inferior vena cava and the venous flow pattern on Doppler ultrasound in the portal, suprahepatic and intrarenal veins (Figure 1), shows a good correlation with renal dysfunction in patients after cardiac surgery,^(10)^ and may be useful in strategies of resuscitation and management of ultrafiltration in patients on hemodialysis,^(11^.^12)^ being an interesting tool for the evaluation of right FT (Figure 2).

IVC - inferior vena cava; US - ultrasound.

CVP - central venous pressure; GEDV - global end-diastolic volume; IVC - inferior vena cava; VExUS - ultrasound of excess venous congestion; EVLW - extravascular pulmonary water; PAOP - pulmonary artery occlusion pressure; US - ultrasound.

## CONCLUSION

The growing evidence in critically ill patients of aggravation of organ dysfunction related to fluid overload implies that hemodynamic evaluations should advance beyond fluid responsiveness and begin to encompass fluid tolerance. The coordinated evaluation of these two variables has the potential to prevent and reverse acute organ dysfunction and assigns a new obligation to intensivists: fluid responsibility.
